# Supplementary material for: SCN4B acts as a metastasis-suppressor gene preventing hyperactivation of cell migration in breast cancer
Source: Nat Commun. 2016 Dec 5;7:13648. doi: 10.1038/ncomms13648 (PMC5150224; doi:10.1038/ncomms13648)
Supplement: Supplementary Information — Supplementary Figures 1-10, Supplementary Table 1, Supplementary References. [file ncomms13648-s1.pdf]

Breast hyperplasia

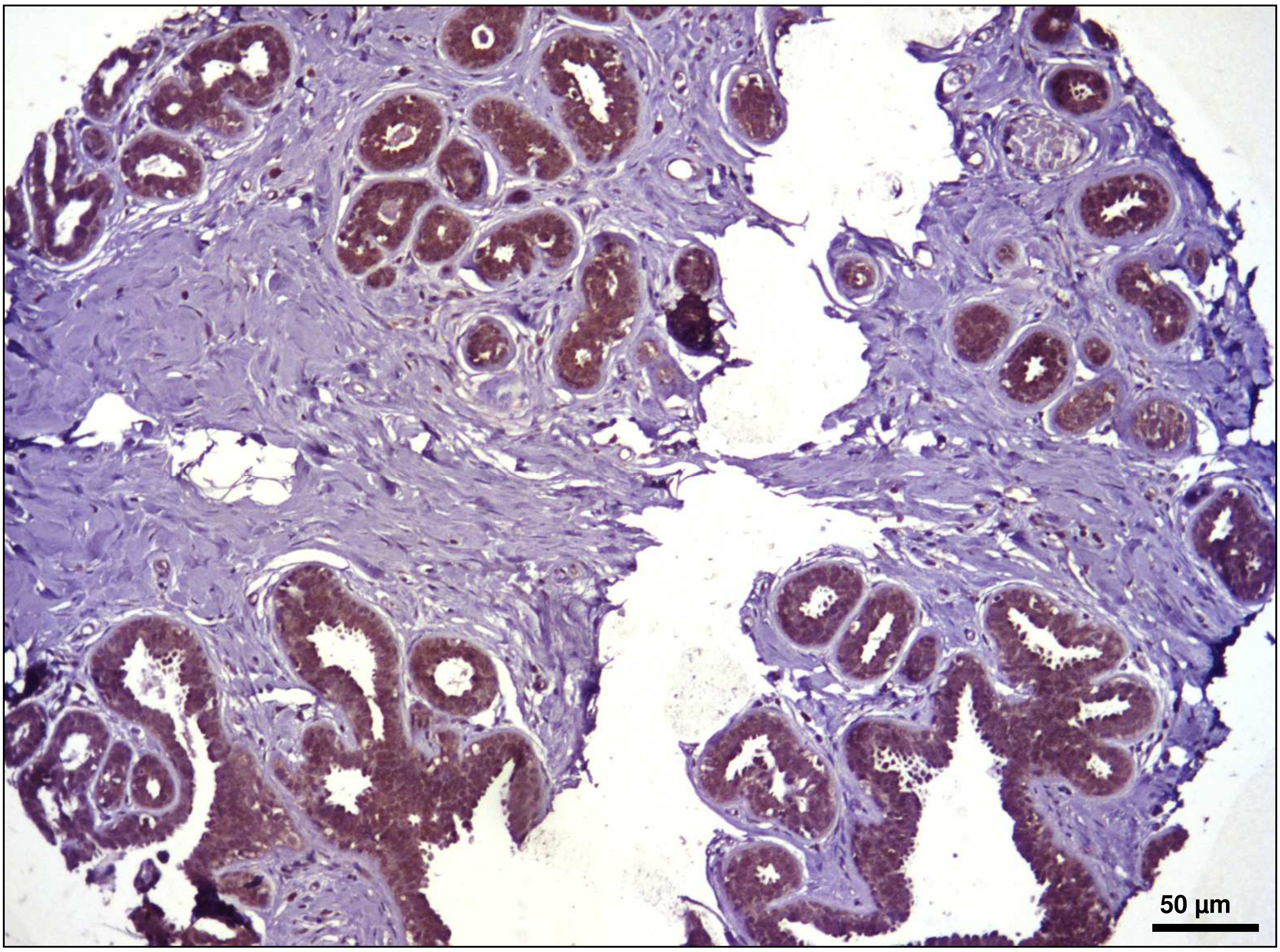

Supplementary Figure 1: *SCN4B*/ β4 expression in human breast hyperplasia. β4 protein (expression of the *SCN4B* gene) was analysed by immunohistochemistry on breast hyperplasia from TMA. Scale bar, 50 µm.

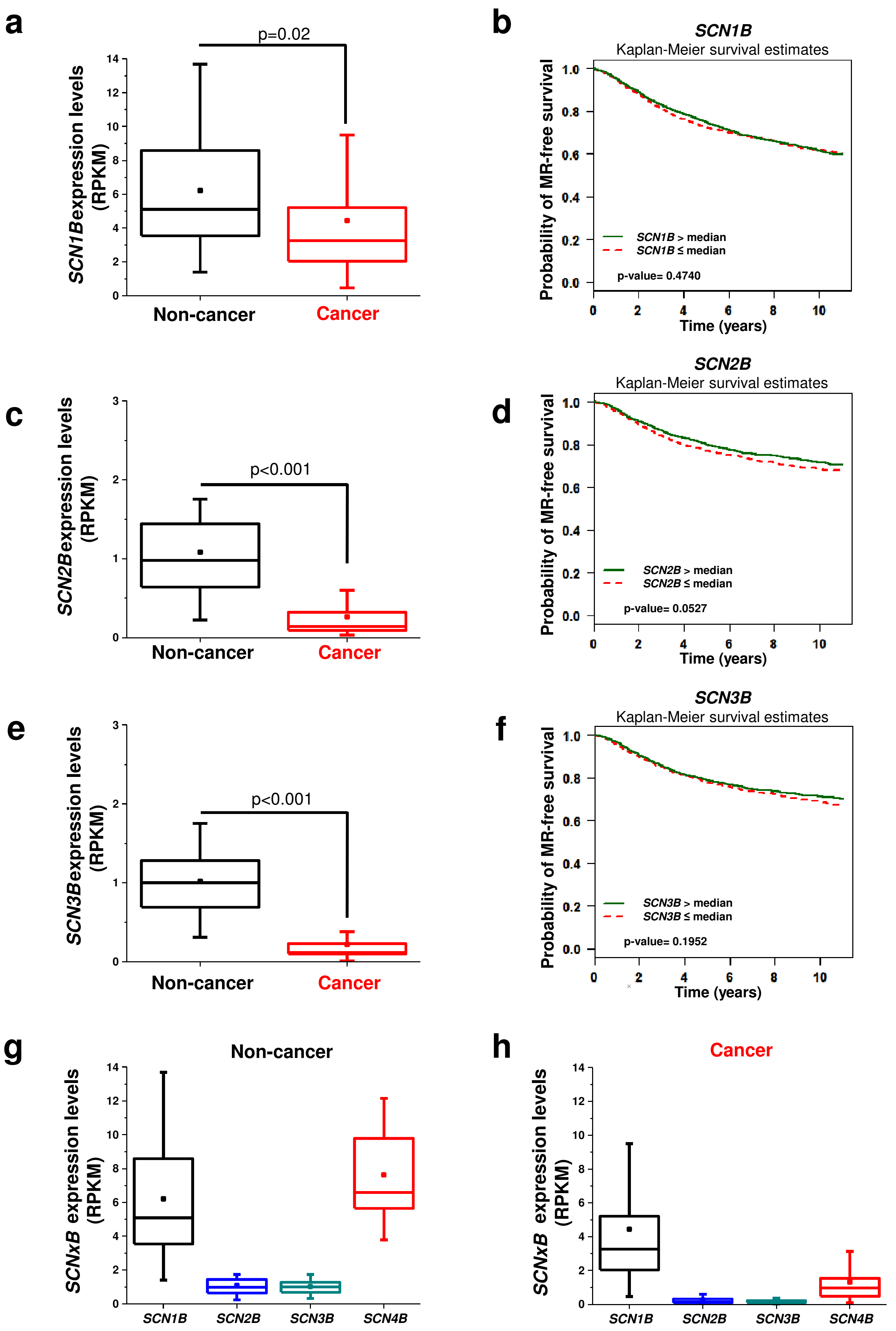

a
p=0.02
SCN1B expression levels (RPKM)
Non-cancer
Cancer
b
SCN1B
Kaplan-Meier survival estimates
Probability of MR-free survival
SCN1B > median
SCN1B ≤ median
p-value= 0.4740
Time (years)
c
p<0.001
SCN2B expression levels (RPKM)
Non-cancer
Cancer
d
SCN2B
Kaplan-Meier survival estimates
Probability of MR-free survival
SCN2B > median
SCN2B ≤ median
p-value= 0.0527
Time (years)
e
p<0.001
SCN3B expression levels (RPKM)
Non-cancer
Cancer
f
SCN3B
Kaplan-Meier survival estimates
Probability of MR-free survival
SCN3B > median
SCN3B ≤ median
p-value= 0.1952
Time (years)
g
Non-cancer
SCNxB expression levels (RPKM)
SCN1B
SCN2B
SCN3B
SCN4B
h
Cancer
SCNxB expression levels (RPKM)
SCN1B
SCN2B
SCN3B
SCN4B

Supplementary Figure 2: *SCNxB* gene expression in human breast cancer tissues and associations with patient survival without metastatic relapse.

a, **c, e**, Expression of the *SCNxB* gene in non-cancer (n= 29) and in breast cancer tissues (n= 145) was analysed using data obtained from The Cancer Genome Atlas (TCGA) and RNA expression is given as reads per kilobase per million (RPKM). Box plots indicate the first quartile, the median, and the third quartile, squares indicate the mean. **a**, *SCN1B* gene was significantly down-regulated in cancer compared to non-cancer tissues (p=0.02). **c**, *SCN2B* gene was significantly down-regulated in cancer compared to non-cancer tissues (p<0.01) and **e**, *SCN3B* gene was also significantly down-regulated in cancer compared to non-cancer tissues (p<0.01). **b, d, f,** Kaplan-Meier analyses of metastatic relapse (MR)-free survival performed on data pooled from cohorts (see Methods) for the expression of **b**, *SCN1B* gene (n= 5,436 patients), **d**, *SCN2B* gene (n=3,826 patients), **f**, *SCN3B* gene (3,751 patients). For these three genes, there was no statistical difference in MR-free survival between groups, expressing the considered *SCNxB* gene highly (> median) or weakly (<median). **g-h**, Analyses of RNA expression levels (expressed in RPKM) of *SCNxB* genes in **g**, non-cancer (n= 29) and in **h**, breast cancer tissues (n= 145), from The Cancer Genome Atlas (TCGA).

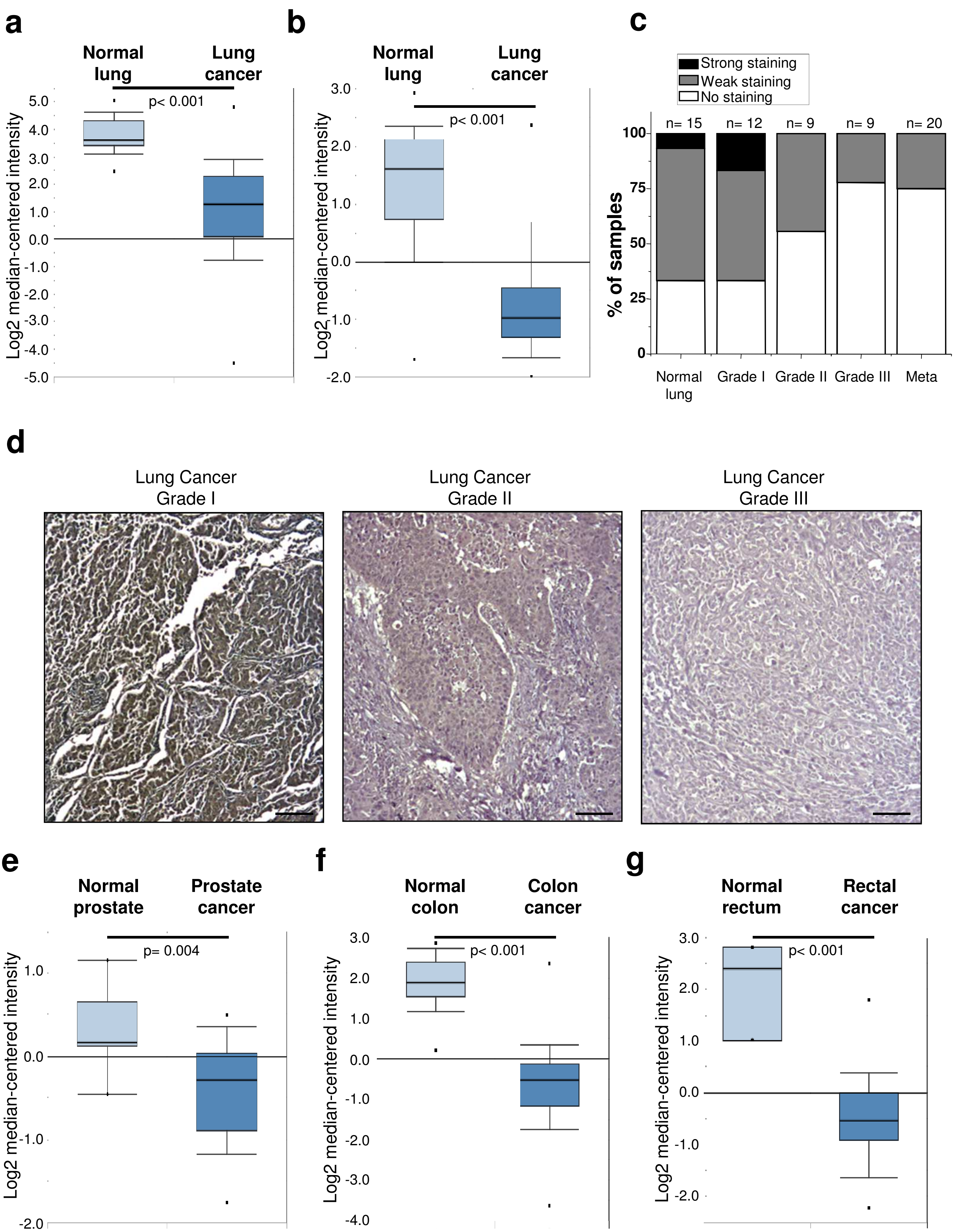

a
Normal lung
Lung cancer
p< 0.001
Log2 median-centered intensity
5.0
4.0
3.0
2.0
1.0
0.0
-1.0
-2.0
-3.0
-4.0
-5.0
b
Normal lung
Lung cancer
p< 0.001
Log2 median-centered intensity
3.0
2.0
1.0
0.0
-1.0
-2.0
c
Strong staining
Weak staining
No staining
% of samples
n= 15
n= 12
n= 9
n= 9
n= 20
100
75
50
25
0
Normal lung
Grade I
Grade II
Grade III
Meta
d
Lung Cancer Grade I
Lung Cancer Grade II
Lung Cancer Grade III
e
Normal prostate
Prostate cancer
p= 0.004
Log2 median-centered intensity
1.0
0.0
-1.0
-2.0
f
Normal colon
Colon cancer
p< 0.001
Log2 median-centered intensity
3.0
2.0
1.0
0.0
-1.0
-2.0
-3.0
-4.0
g
Normal rectum
Rectal cancer
p< 0.001
Log2 median-centered intensity
3.0
2.0
1.0
0.0
-1.0
-2.0

Supplementary Figure 3: *SCN4B* gene down-regulation in human cancer tissues. The expression level of the *SCN4B* gene was assessed from published data, reporting information on 19,574 genes with the Affymetrix Human Genome U133 Plus 2.0 Array, in normal lung and in lung adenocarcinomas**.** Normalization and statistical analysis were performed using standard settings: for each array, data were log2-transformed and median centered**.** **a**, In the Okayama *et al.* lung cancer study group [1], there was a significant reduction ($p<0.001$) of *SCN4B* expression in lung adenocarcinoma tissues (n=226) compared to normal lung tissues (n=20). **b**, In the Hou *et al.* Non-small-cell Lung cancer study [2], there was a significant reduction ($p<0.001$) of *SCN4B* expression in lung adenocarcinoma tissues (n=45) compared to normal lung tissues (n=65). **c**, the protein (β4) expression of the *SCN4B* gene was analysed by immunohistochemistry on tissues arrays containing normal lung and lung cancer (from grade I to grade III) tissues, as well as metastases (Meta) samples. β4 staining was analysed and samples were stratified in “no staining”, “weak staining” or “strong staining” groups. Diagrams indicate the proportion of samples showing no (white), weak (gray) or strong (black) β4 staining in normal and cancer lung samples. The number of samples per condition is indicated between brackets. There was a tendency, yet not statistically different, for a reduced expression of β4 in high-grade lung adenocarcinomas. **d**, Representative β4 staining pictures from grade I, grade II and grade III lung adenocarcinoma. Scale bars, 50 µm. **e**, The expression level of the *SCN4B* gene was assessed in normal and prostate adenocarcinomas [3]. Gene expression was assessed using Affymetrix GeneChip U133 array (Plus 2.0 chip) consisting of >52,000 transcripts from whole human genome transcripts. There was a significant reduction ($p=0.004$) of *SCN4B* expression in prostate carcinomas (n=13) compared to normal prostate gland (n=8) and. **f**, Using the TCGA colorectal data for RNA sequencing, the *SCN4B* expression was analysed and there was a significant reduction ($p<0.001$) of *SCN4B* expression in prostate carcinomas (n=101) compared to normal colon (n=19). **g**, Similarly, the *SCN4B* expression was significantly reduced ($p<0.001$) in rectal carcinomas (n=60) compared to normal rectum (n=3).

Normal Oesophagus

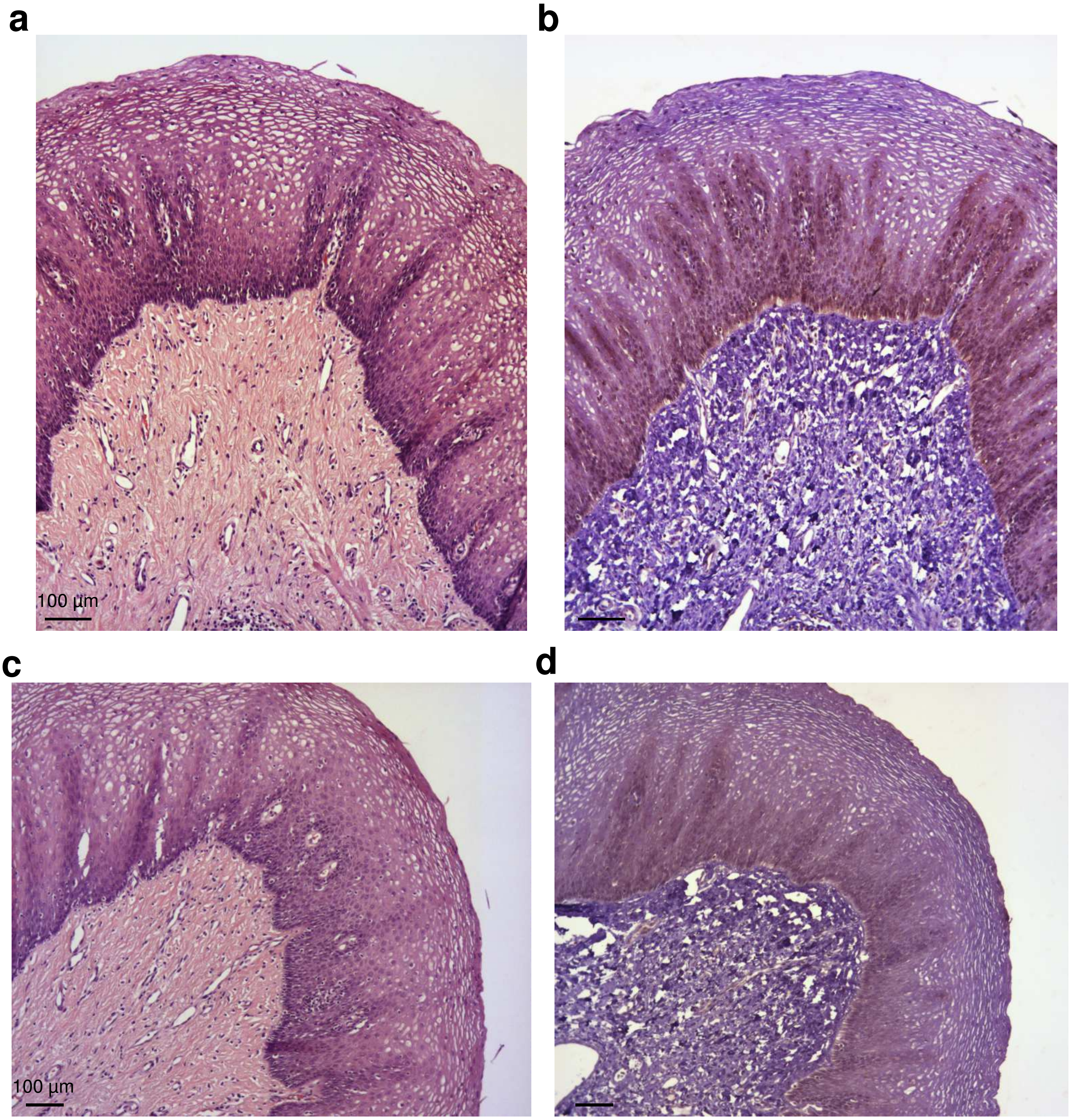

Supplementary Figure 4: *SCN4B* protein expression (β4) in normal oesophagus.
Two normal human oesophagus samples were stained with haematoxylin and eosin (**a, c**) and protein expression of β4 was analysed by immunohistochemistry (**b, d**).

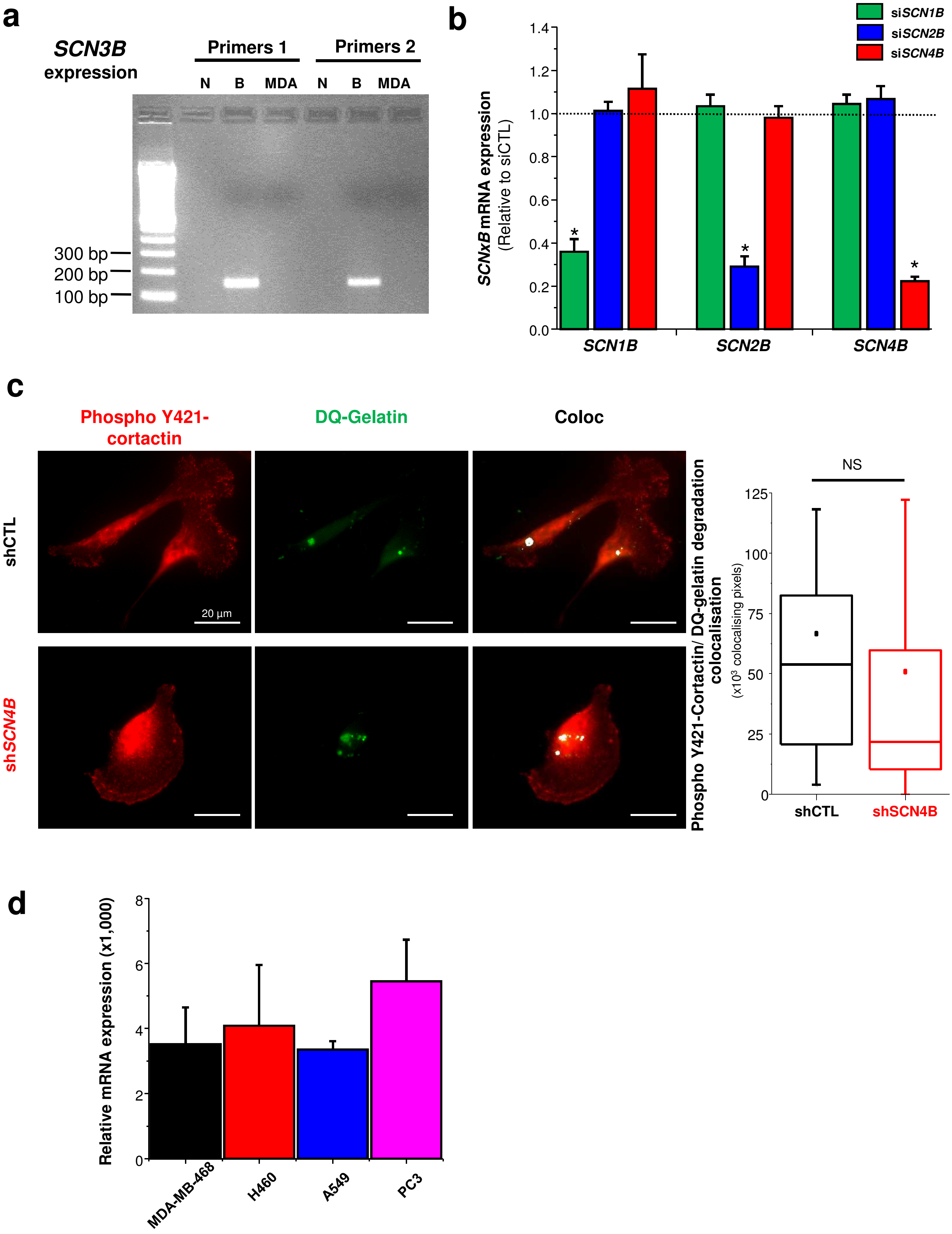

a
SCN3B expression
Primers 1
Primers 2
N B MDA N B MDA
300 bp
200 bp
100 bp
b
siSCN1B
siSCN2B
siSCN4B
SCNxB mRNA expression (Relative to siCTL)
1.4
1.2
1.0
0.8
0.6
0.4
0.2
0.0
SCN1B
SCN2B
SCN4B
c
Phospho Y421-cortactin
DQ-Gelatin
Coloc
shCTL
shSCN4B
20 µm
NS
Phospho Y421-Cortactin/ DQ-gelatin degradation colocalisation
(x10³ colocalising pixels)
125
100
75
50
25
0
shCTL
shSCN4B
d
Relative mRNA expression (x1,000)
8
6
4
2
0
MDA-MB-468
H460
A549
PC3

Supplementary Figure 5:

a, Expression of *SCN3B* gene mRNA was assessed in MDA-MB-231-Luc cells (MDA) by conventional PCR using two couples of primers having the following sequences: Couple of primers 1: forward primer, 5'-GGCTGATCCCCCTAAGAGTC-3'; reverse primer 5'-CGGCTTTTGAGACCTTTCTG-3' (expected size of the amplicon: 147bp) and couple of primers 2: forward primer, 5'-GAGGGCGGTAAAGATTTCCT-3'; reverse primer 5'-AGAGGCCAGAGTCGTTCAGA-3' (expected size of the amplicon: 154bp). Human brain total RNA extracts (B, Ref 636530, Ozyme) were used as positive controls. N represent negative controls performed in the absence of cDNA template. **b**, The expression of *SCN1B, SCN2B and SCN4B* gene mRNA was assessed by quantitative PCR in MDA-MB-231-Luc cells transfected with specific silencing RNA targeting *SCN1B (*si*SCN1B), SCN2B (*si*SCN2B), SCN4B (*si*SCN4B),* or a control sequence targeting no identified *gene (siCTL). Results are expressed as* relative to that of HPRT-1 gene expression and to corresponding genes in siCTL conditions. These results come from 4-6 independent experiments. *, indicates as statistical difference at $p<0.05$ compared to siCTL condition.**c**, Left panel, Immunofluorescence imaging of the actin nucleation promoting factor phospho (Y421)-cortactin (red fluorescence) performed on shCTL and sh*SCN4B* breast cancer cells grown on a film of Matrigel™ containing DQ-gelatin® as a fluorogenic substrate, that when degraded by proteases emit green fluorescence. Co-localisation areas of red and green fluorescence (Coloc, white pixels) show focalised areas of matrix degradation that are representative of invadopodia activity. Right panel, co-localisation pixels were quantified per cells. The analysis represents results obtained from 99 and 69 cells for shCTL and sh*SCN4B*, respectively. NS stands for no statistical difference. **d,** The expression of the *SCN4B* gene (mRNA) was studied by RT- quantitative PCR in human breast MDA-MB-468, non-small cell lung H460, non-small cell lung A549 and prostate PC3 cancer cell lines. Results are given as the amount of mRNA relative to that of HPRT-1 gene expression (n= 3-11 separate experiments).

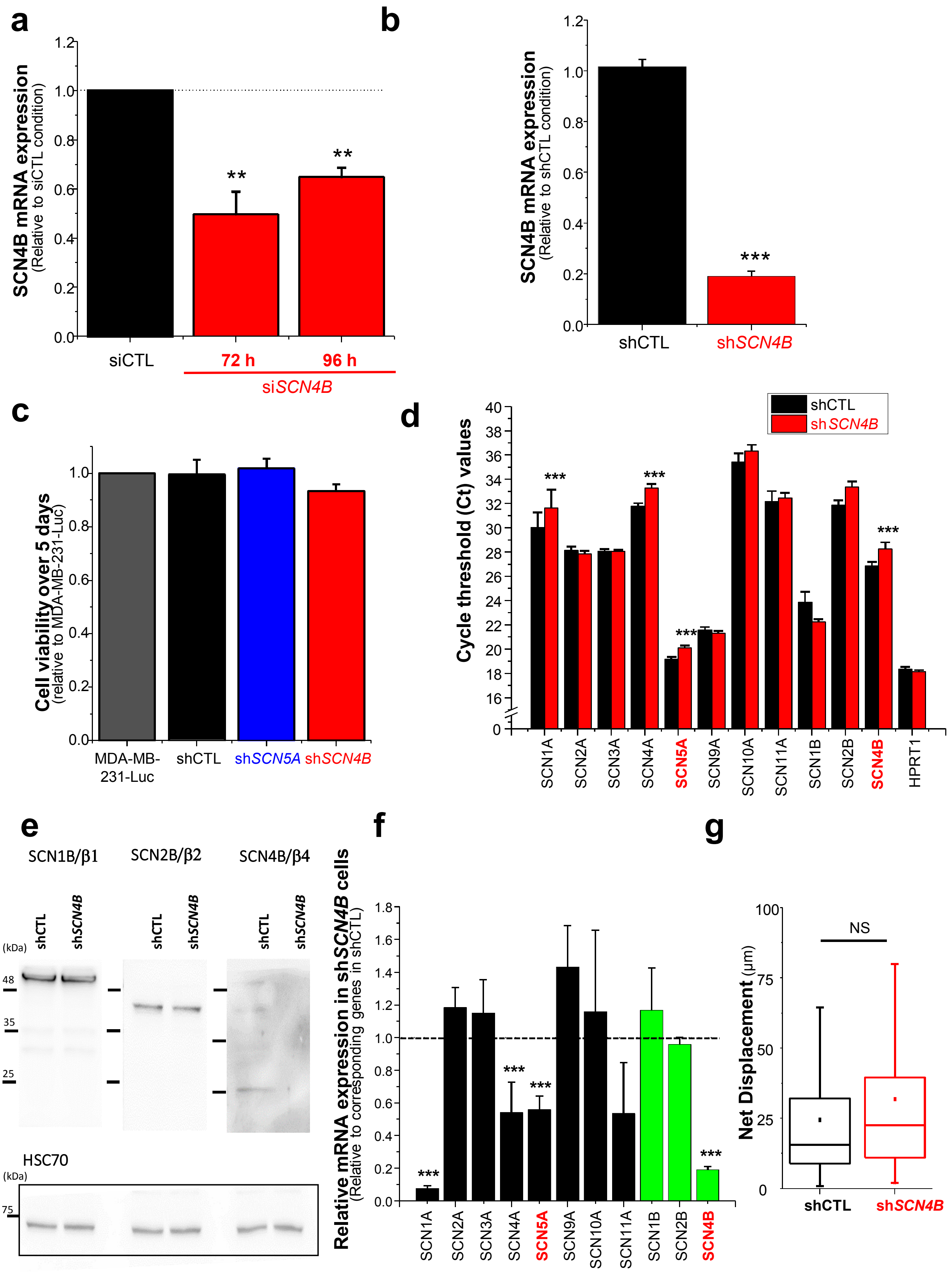
a
SCN4B mRNA expression
(Relative to siCTL condition)
1.2
1.0
0.8
0.6
0.4
0.2
0.0
**
**
siCTL
72 h
96 h
siSCN4B
b
SCN4B mRNA expression
(Relative to shCTL condition)
***
shCTL
shSCN4B
c
Cell viability over 5 days
(relative to MDA-MB-231-Luc)
MDA-MB-231-Luc
shCTL
shSCN5A
shSCN4B
d
Cycle threshold (Ct) values
shCTL
shSCN4B
***
SCN1A
SCN2A
SCN3A
SCN4A
SCN5A
SCN9A
SCN10A
SCN11A
SCN1B
SCN2B
SCN4B
HPRT1
e
SCN1B/β1
SCN2B/β2
SCN4B/β4
(kDa)
48
35
25
HSC70
75
f
Relative mRNA expression in shSCN4B cells
(Relative to corresponding genes in shCTL)
g
Net Displacement (μm)
NS
100
75
50
25
0

Supplementary Figure 6:

a, Expression of *SCN4B* gene mRNA in MDA-MB-231-Luc cells transfected with siCTL and si*SCN4B*. Results are given as the amount of mRNA relative to that of HPRT-1 gene expression, 72 h and 96 h after transfection, and expressed as a ratio of the siCTL condition at the corresponding day post-transfection. From 5 independent experiments. ** indicates a statistical difference from siCTL at p<0.01. **b**, Expression of *SCN4B* gene mRNA in shCTL and sh*SCN4B* cells. Results are given as the amount of mRNA relative to that of HPRT-1 gene expression, and expressed as a ratio of the shCTL condition, from 37 independent real-time PCR experiments. *** indicates a statistical difference from shCTL at p<0.001. **c**, Comparison of MDA-MB-231-Luc, shCTL, sh*SCN5A* and sh*SCN4B* cell growth and viability measured by the MTT assay after 5 days culture, expressed relative to the MDA-MB-231-Luc cell line (n= 3 independent experiments). **d**, mRNA expression of the pore-forming *SCNxA* and auxiliary *SCNxB* subunits gene was assessed by real-time PCR in shCTL and sh*SCN4B* cells, and expressed as Cycle Threshold values (Ct). Data for*SCN8A* and *SCN3B* are not indicated in the graph because their gene expression was undetectable after 40 amplification cycles. These results come from 3-6 independent experiments. *** indicates a statistical difference from the corresponding gene expression in shCTL cells at p<0.001. **e**, SCN1B/β1, SCN2B/β2 and SCN4B/β4 protein expression in shCTL and sh*SCN4B* cells assessed by western blotting experiments. HSC70 was used as a loading control. The blot is representative of 4 independent experiments. **f**, Relative mRNA expression of the pore-forming *SCNxA* and auxiliary *SCNxB* subunits gene was assessed by real-time PCR in sh*SCN4B* cells compared to shCTL (ΔΔCt). Data for*SCN8A* and *SCN3B* are not indicated in the graph because their gene expression was undetectable after 40 amplification cycles. These results come from 3-6 independent experiments. *** indicates a statistical difference from the corresponding gene expression in shCTL cells at p<0.001. **g**, The net displacement (in µm) from migrating cancer cell was analyzed after 180 min in shCTL and sh*SCN4B* cell lines from time-lapse experiments and results shown were obtained from 106 and 96 cells, respectively. NS stands for no statistical difference.

sh*SCN4B*

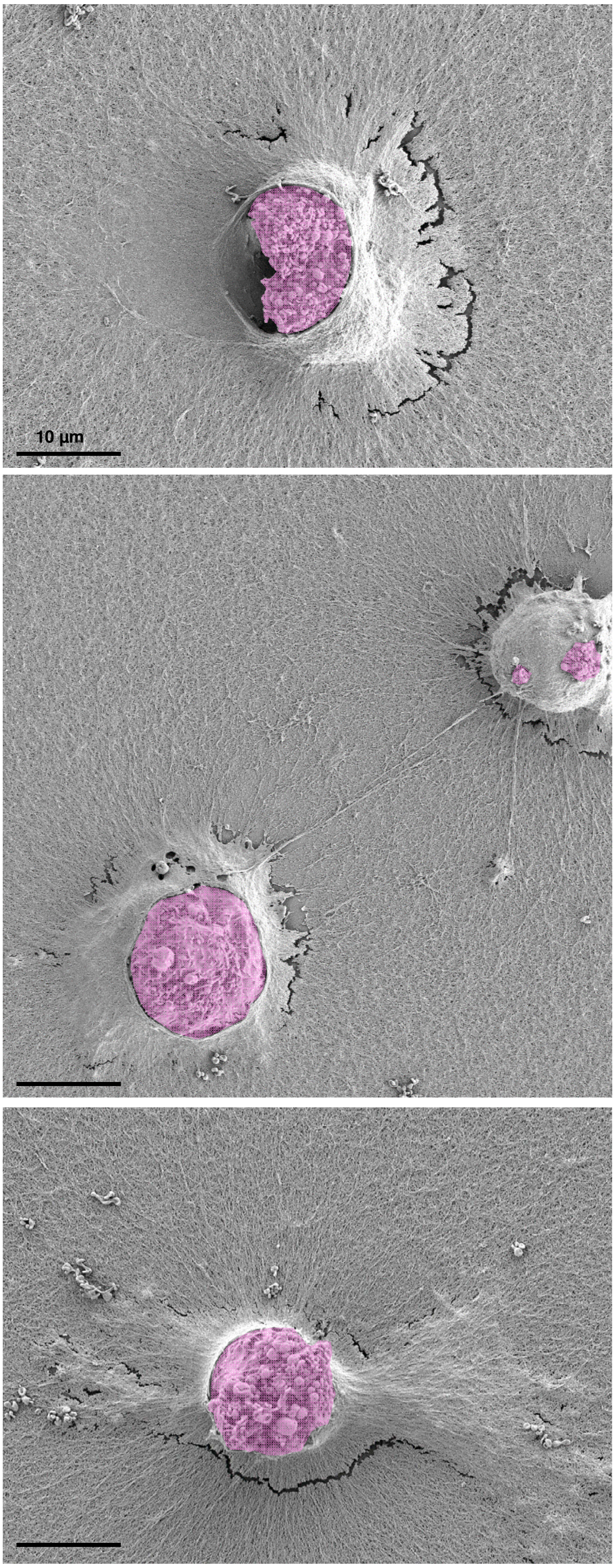

Supplementary Figure 7:
Scanning electron microscopy observations, 24 hr after cell seeding, of sh*SCN4B* cell invasion through Matrigel™ (4 mg/mL) coated on the glass coverslip. Scale bars, 10 µm.

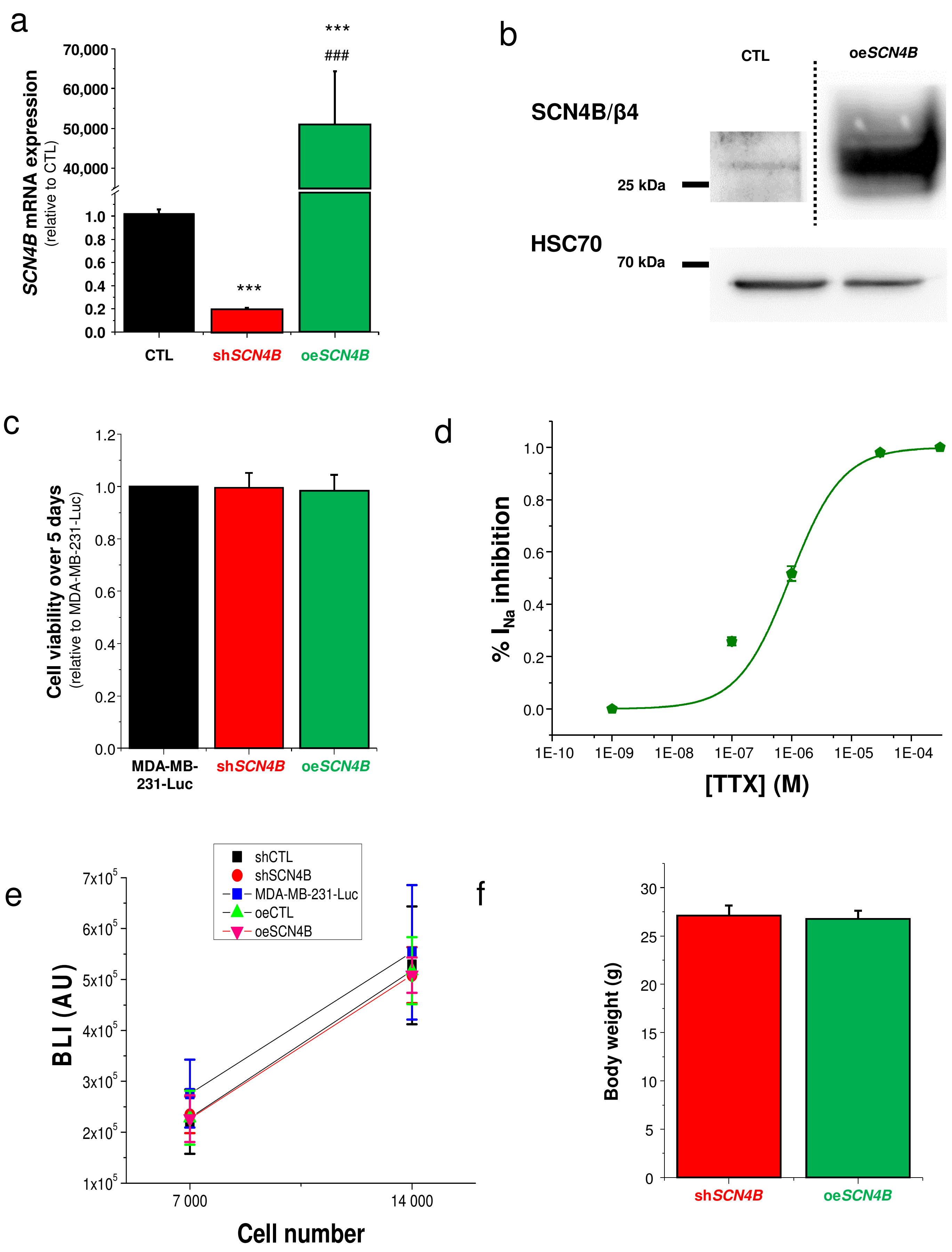
a
***
###
70,000
60,000
50,000
40,000
1.0
0.8
0.6
0.4
0.2
0.0
SCN4B mRNA expression
(relative to CTL)
***
CTL
shSCN4B
oeSCN4B
b
CTL
oeSCN4B
SCN4B/β4
25 kDa
HSC70
70 kDa
c
Cell viability over 5 days
(relative to MDA-MB-231-Luc)
1.2
1.0
0.8
0.6
0.4
0.2
0.0
MDA-MB-231-Luc
shSCN4B
oeSCN4B
d
% I_Na inhibition
1.0
0.8
0.6
0.4
0.2
0.0
1E-10
1E-09
1E-08
1E-07
1E-06
1E-05
1E-04
[TTX] (M)
e
shCTL
shSCN4B
MDA-MB-231-Luc
oeCTL
oeSCN4B
BLI (AU)
7x10^5
6x10^5
5x10^5
4x10^5
3x10^5
2x10^5
1x10^5
7 000
14 000
Cell number
f
Body weight (g)
30
25
20
15
10
5
0
shSCN4B
oeSCN4B

Supplementary Figure 8:

a, Relative mRNA expression of *SCN4B* gene, assessed by real-time PCR in CTL, sh*SCN4B* and oe*SCN4B* cells, expressed as a ratio of *HPRT1* gene expression and relative to the expression in CTL cells. Results are coming from 18 independent experiments. *** indicates a statistical difference from CTL cells at p<0.001, and ### indicates a statistical difference from sh*SCN4B* cells at p<0.001. **b**, Western blot experiments showing high expression of SCN4B/β4 protein in oeSCN4B compared to CTL cells. HSC70 (70 kDa) was used as a loading control. Blots shown here were obtained in the same film with different exposure time for oeSCN4B (1 s) compared to CTL (30s). **c**, Comparison of MDA-MB-231-Luc, sh*SCN4B* and oe*SCN4B* cell growth and viability measured by the MTT assay after 5 days culture, expressed relative to the MDA-MB-231-Luc cell line (n= 3 independent experiments). **d**, Dose-response effect of TTX on the inhibition of I_{Na} peak elicited by a membrane depolarization from -100 to -5 mV in oe*SCN4B* cells (n=8). Data were fitted with the Hill equation giving an IC_{50} value of 0.95±0.30. **e**, MDA-MB-231-Luc-derived cell lines were seeded at different densities (7,000 and 14,000 cells/well of a 96-well plate). After 24hr growth, cells were lysed and the bioluminescent signal (BLI) was measured after the addition of luciferin (Luciferase Assay System kit, Promega) and expressed as arbitrary units (AU). Results are expressed as mean ± SEM. There was no significant difference between the 5 conditions tested (MDA-MB-231Luc, shCTL, sh*SCN4B*, oeCTL, oe*SCN4B*). **f**, Mice tail vein-injected with shSCN4B (n=7) or oeSCN4B (n=8) cells showed identical body weight (g) at the completion of the study (9 weeks after cell injection).

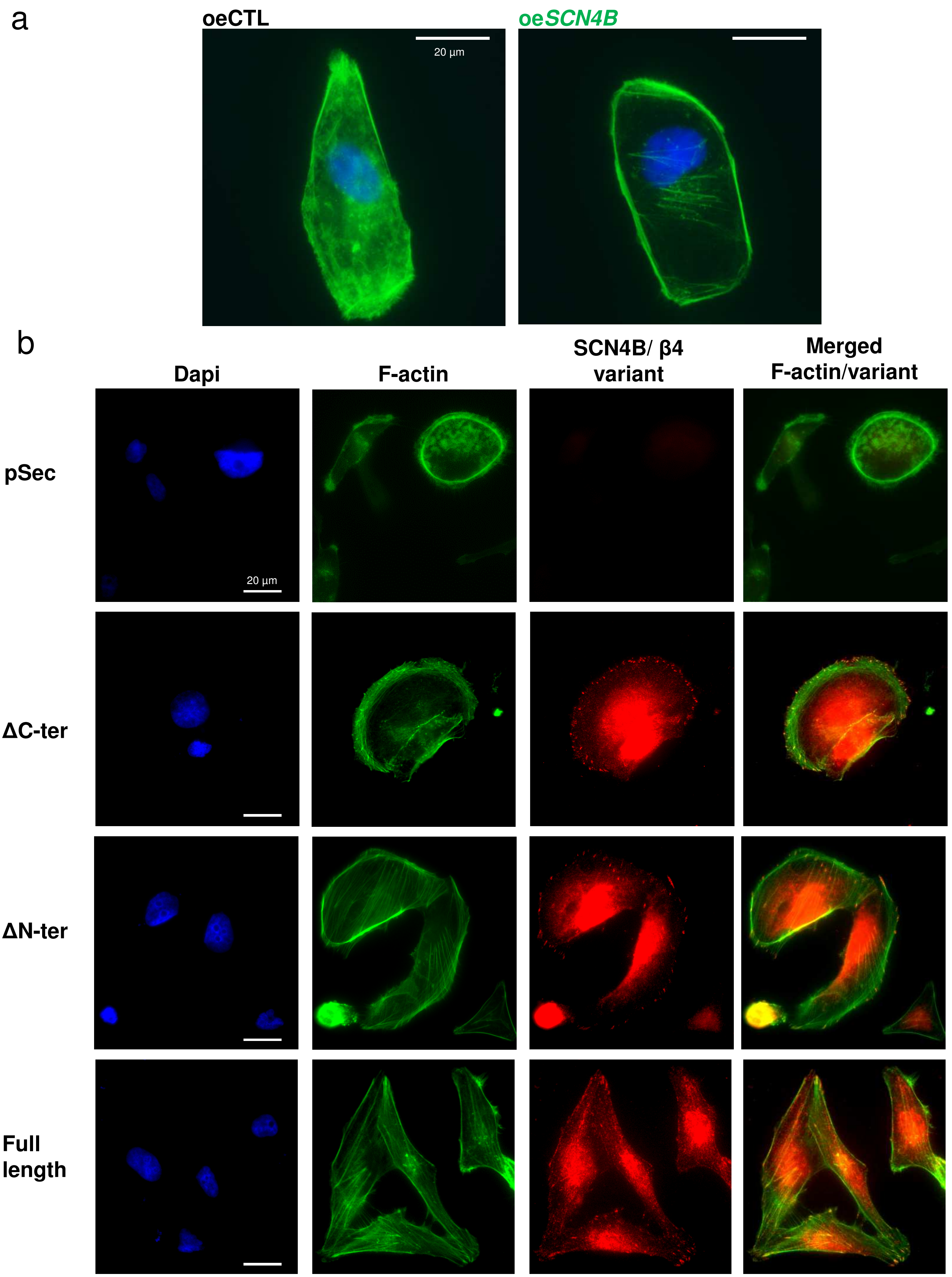
a
oeCTL
oeSCN4B
20 µm
b
Dapi
F-actin
SCN4B/ β4 variant
Merged F-actin/variant
pSec
ΔC-ter
ΔN-ter
Full length
20 µm

Supplementary Figure 9:
a, Representative images of oeCTL and oe*SCN4B* cells for which the F-actin cytoskeleton was stained with phalloidin-AlexaFluor488 (green) and nuclei with DAPI (blue). Scale bar, 20 µm. **b,** Representative epifluorescence images of sh*SCN4B,* 48 h after their transfection with an empty expression vector (pSec), or with one of the three SCN4B/β4 variants "ΔN-ter", "ΔC-ter" or "Full-length" encoding sequences. F-actin was labelled with phalloidin-AlexaFluor488 (green), nuclei with DAPI (blue), and the different SCN4B/β4 variants were labelled with a primary rabbit anti-His-Tag antibody, and a secondary goat anti-rabbit Texas Red antibody. Scale bar, 20 µm.

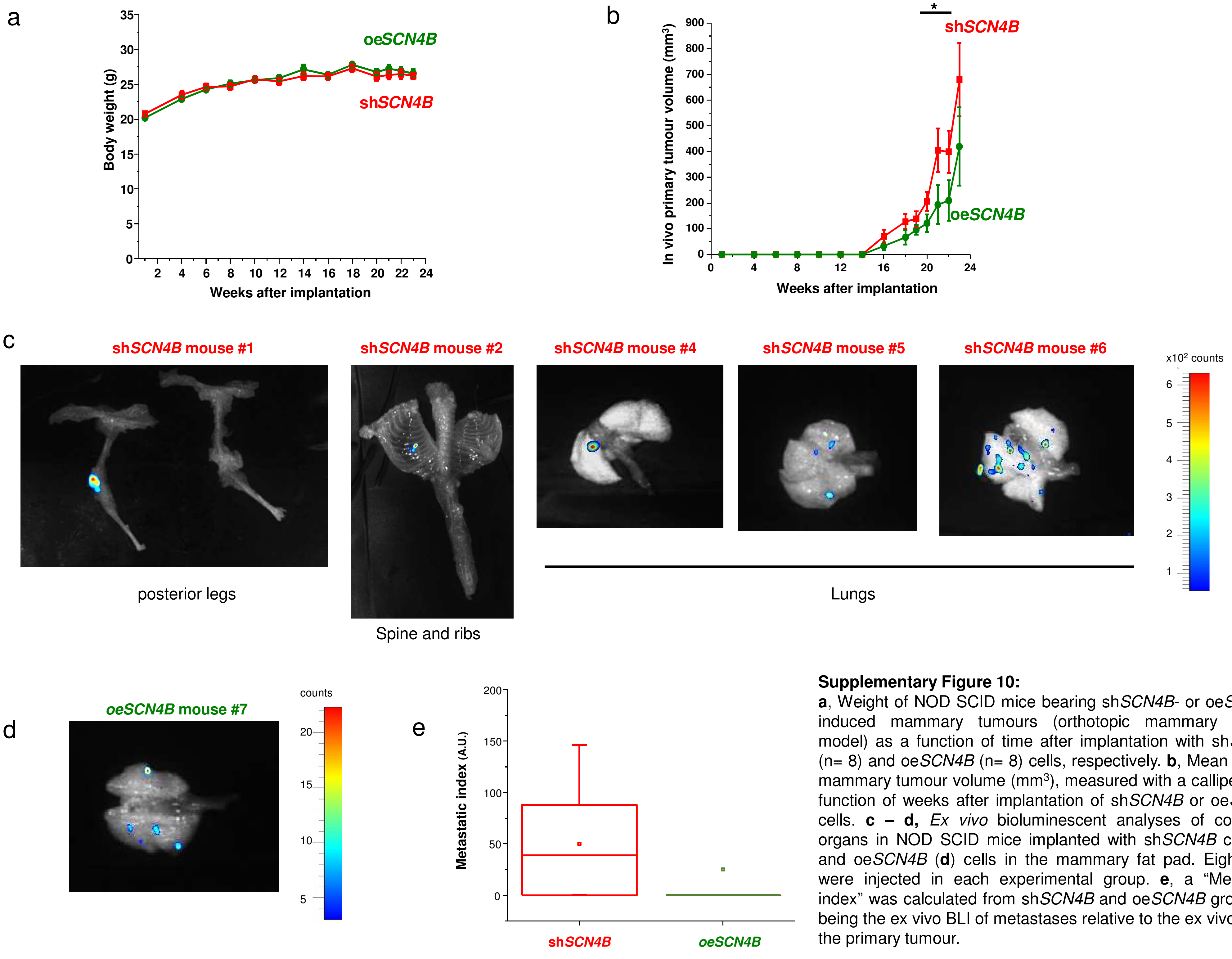

Supplementary Figure 10:
a, Weight of NOD SCID mice bearing sh*SCN4B*- or oe*SCN4B*-induced mammary tumours (orthotopic mammary tumour model) as a function of time after implantation with sh*SCN4B* (n= 8) and oe*SCN4B* (n= 8) cells, respectively. **b**, Mean ± SEM mammary tumour volume (mm^3), measured with a calliper, as a function of weeks after implantation of sh*SCN4B* or oe*SCN4B* cells. **c – d,** *Ex vivo* bioluminescent analyses of colonized organs in NOD SCID mice implanted with sh*SCN4B* cells (**c**) and oe*SCN4B* (**d**) cells in the mammary fat pad. Eight mice were injected in each experimental group. **e**, a "Metastatic index" was calculated from sh*SCN4B* and oe*SCN4B* groups as being the ex vivo BLI of metastases relative to the ex vivo BLI of the primary tumour.

Blots shown in Fig 3b

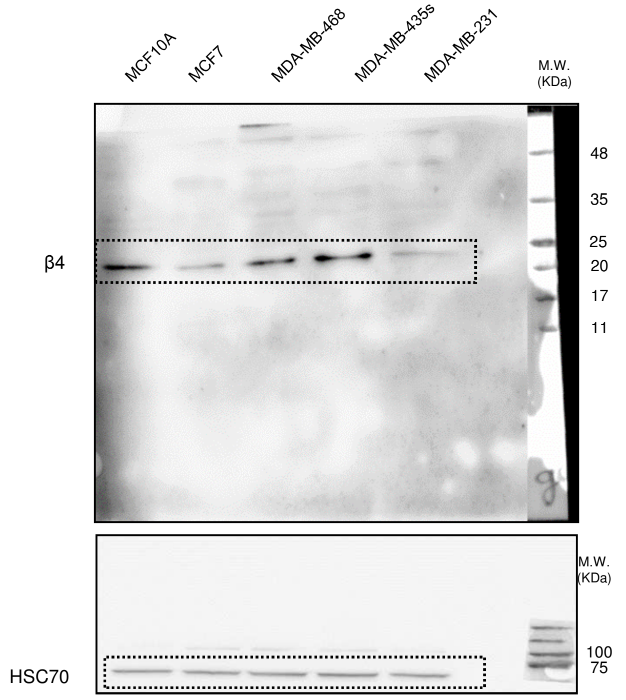

Blots shown in Fig 3d

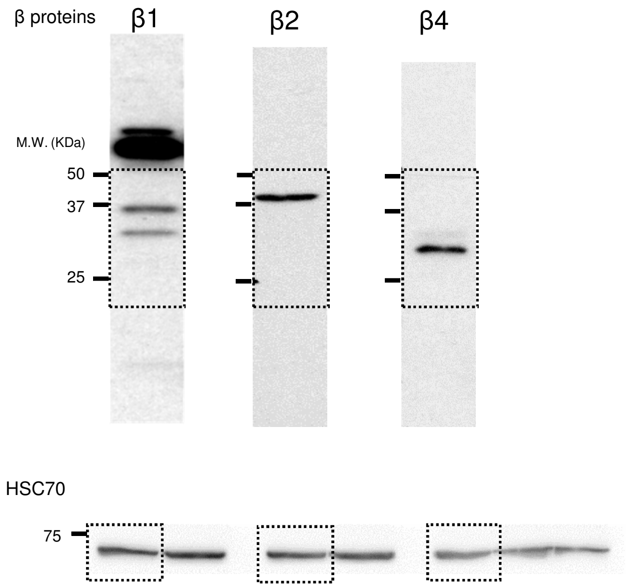

Blots shown in Fig 3e

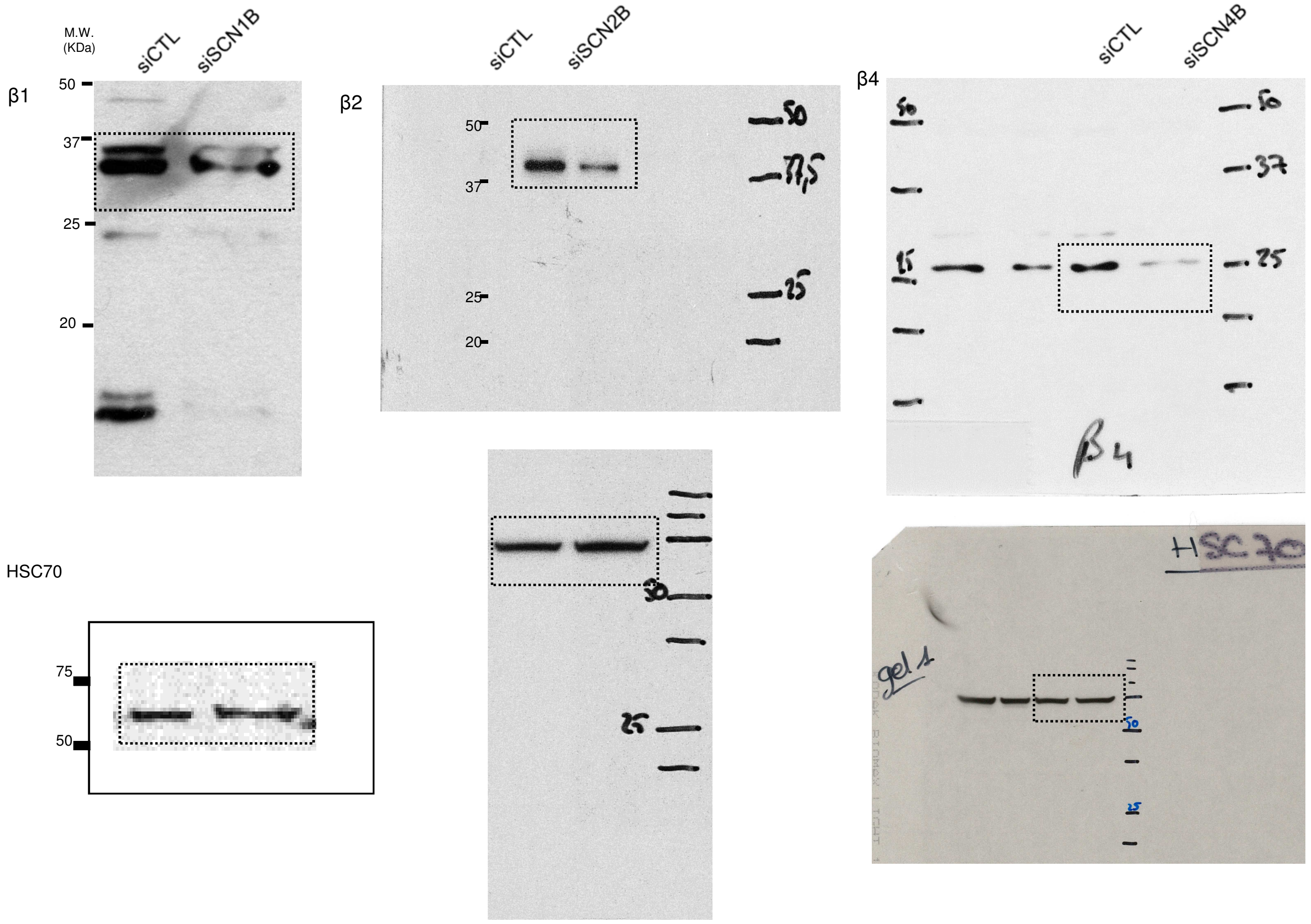

Blots shown in Fig 7i

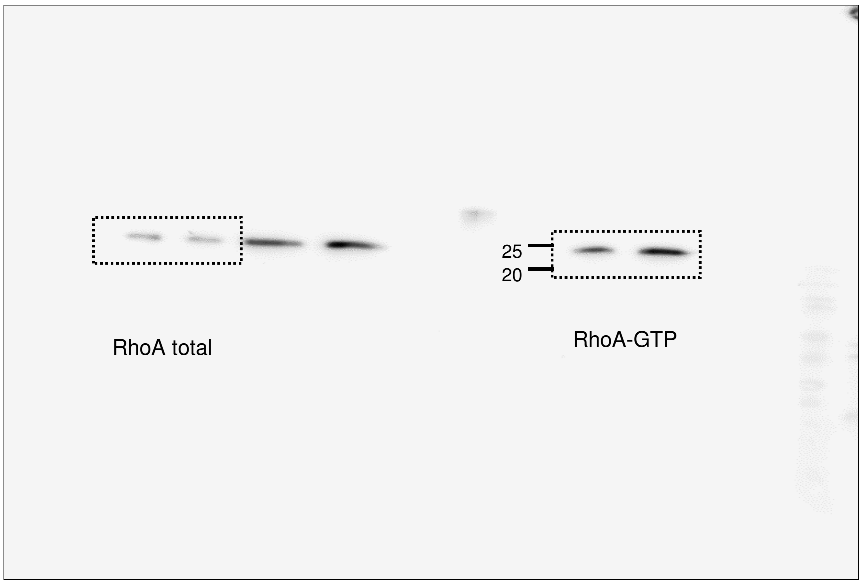

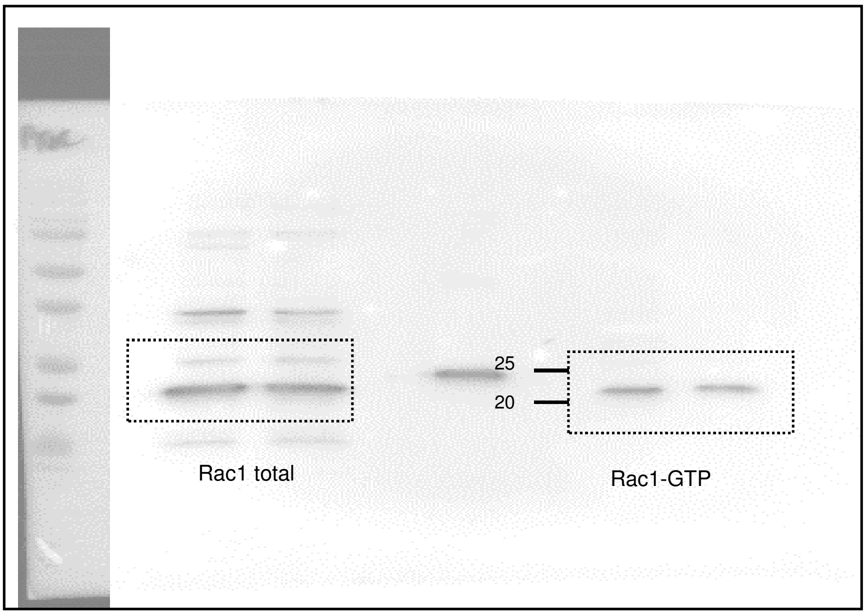

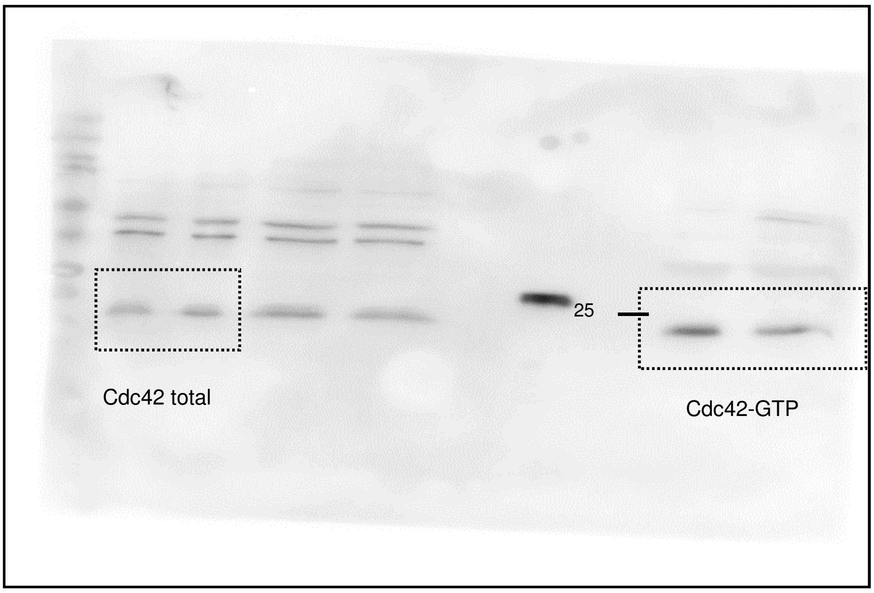

Blots
shown in Fig 8j

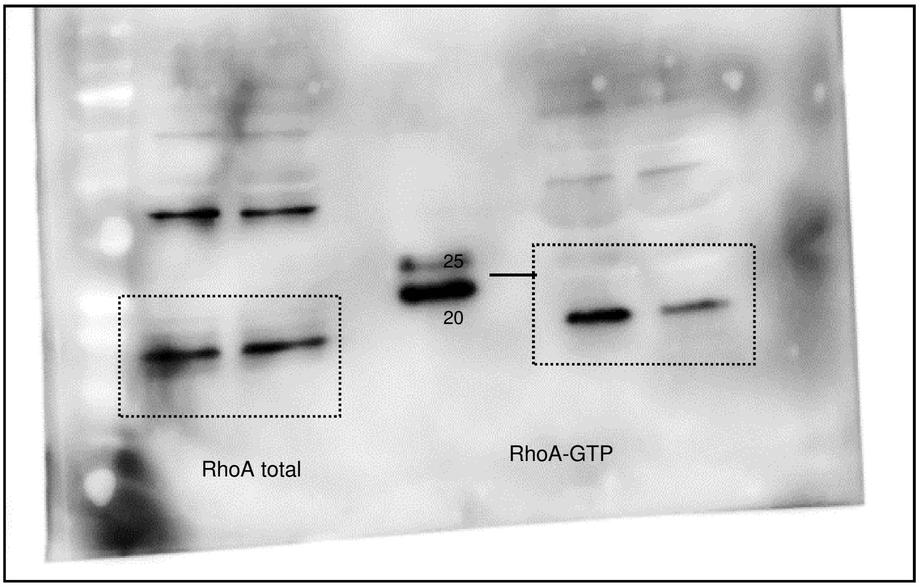

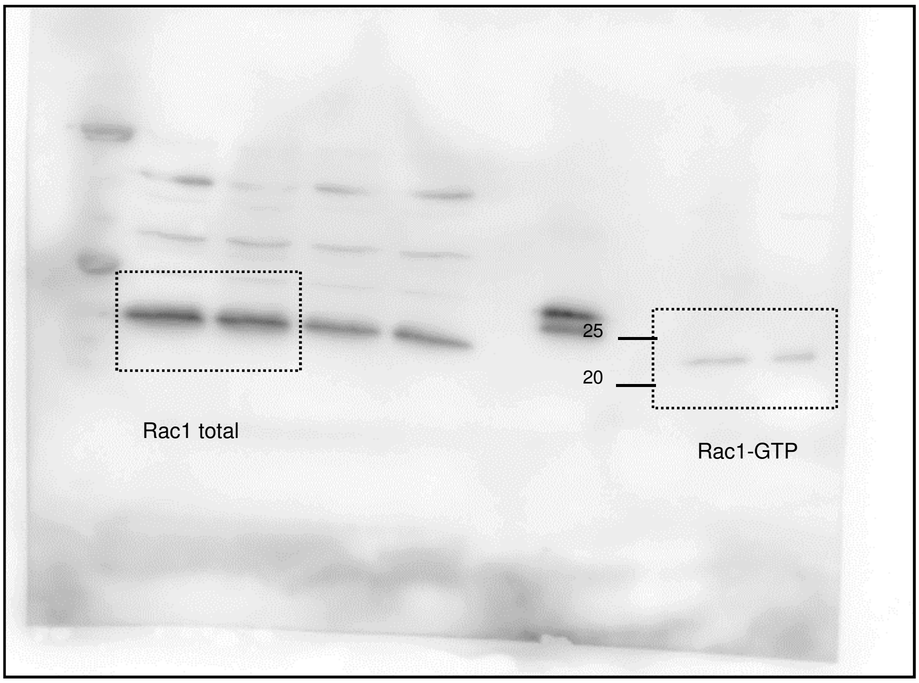

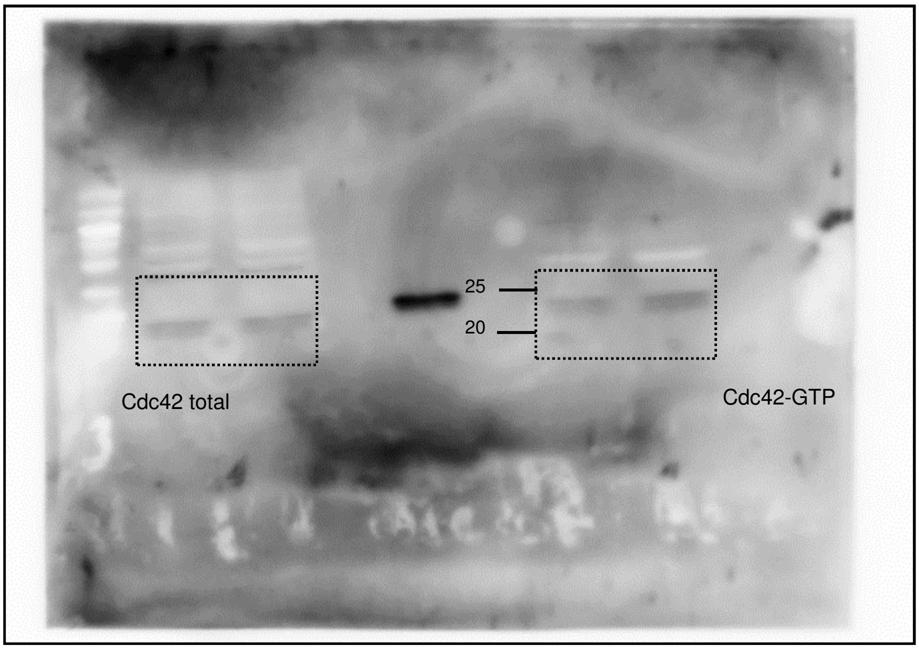

Supplementary figure 11:
Full scans of western blots shown in figures 3, 7 and 8.

Supplementary Table I: PCR primer sequences and expected amplicon sizes (bp)

**Gene**	**protein**	**Forward primers (5' – 3')**	**Reverse primers (5' – 3')**	**Expected size (bp)**
SCN1A	$Na_V1.1$	TTCATGGCTTCCAATCCTTC	TAGCCCCACCTTTGATTTTG	178
SCN2A	$Na_V1.2$	GCCAGCTTATCAATCCCAAA	TCTTCTGCAATGCGTTGTTC	192
SCN3A	$Na_V1.3$	CAAAGGGAAGATCTGGTGGA	AAAGGCCAATGCACCACTAC	115
SCN4A	$Na_V1.4$	TCAACAACCCCTACCTGACC	ACGGACGAGTTCCCATCATA	148
SCN5A	$Na_V1.5$	CACGCGTTCACTTTCCTTC	CATCAGCCAGCTTCTTCACA	208
SCN8A	$Na_V1.6$	CGCCTTATGACCCAGGACTA	GTGCCTCTTCCTGTTGCTTC	247
SCN9A	$Na_V1.7$	GGCTCCTTGTTTTCTGCAAG	TGGCTTGGCTGATGTTACTG	196
SCN10A	$Na_V1.8$	ACCTGGTGGTGCTTAACCTG	TGCTGAAGAAGCTGCAAAGA	168
SCN11A	$Na_V1.9$	CTGTGGTCCTGGTCATTGTG	TGCATTCGCTTCTTGCATAC	233
SCN1B	β1	TGCGCTATGAGAATGAGGTG	GAAGAAGAGCAGGCGGTAGA	176
SCN2B	β2	GTACGATGTGTCGGTGATGC	AGATGACCACAGCCAGGAAG	203
SCN3B	β3	GAGGGCGGTAAAGATTTCCT	AGAGGCCAGAGTCGTTCAGA	154
SCN4B	β4	GAAGTCTGACCCCAAGGTGA	CACATGGCAGGTGTATTTGC	139
HPRT1	Hprt1	TTGCTGACCTGCTGGATTAC	TATGTCCCCTGTTGACTGGT	119
